# Simultaneous Spectrophotometric Estimation of Tenofovir Disoproxil Fumarate and Lamivudine in Three Component Tablet Formulation Containing Efavirenz

**DOI:** 10.4103/0250-474X.73926

**Published:** 2010

**Authors:** R. Sharma, K. Mehta

**Affiliations:** School of Pharmacy, Devi Ahilya University, Takshshila Campus, Khandwa Road, Indore-452 001, India

**Keywords:** Derivative spectroscopy, efavirenz, lamivudine., multicomponent analysis, simultaneous equation method, tenofovir disoproxil fumarate

## Abstract

Three UV spectrophotometric methods have been developed, simultaneous equation method, multicomponent analysis (II) and derivative spectroscopy method (III). The absorption maxima of the drugs were found to be 247, 259 and 272 nm, respectively for efavirenz, tenofovir disoproxil fumarate and lamivudine in methanol:water (50:50) solvent system. Efavirenz, tenofovir disoproxil fumarate and lamivudine obeyed Beer’s law in the concentration range of 10-60, 5-30 and 5-30 μg/ml, respectively. Results of analysis for all the three methods were analyzed and validated for various parameters according to ICH guidelines.

Efavirenz (EFV), (4S)-6-chloro-4-(cyclopropylethynyl) -4-(trifluromethyl)-1-4-dihydro-2H-3,1-benzoxazin-2-one, is an antiretroviral drug which is a non-nucleoside reverse transcriptase inhibitor (NNRTI)[1,2]. EFV has been determined by UV spectroscopic[3] and RP-HPLC[4] methods in single and in combined dosage form. Tenofovir disoproxil fumarate (TDF), 9-((R)-2-((bis(((isopropoxycarbonyl)oxy)methoxy)phosphinyl)methoxy)propyl)adenine fumarate (1:1), is a nucleotide analogue reverse transcriptase inhibitor (nRTIs)[1,2]. TDF has been determined in spiked human plasma by HPLC[5,6]. The estimation of TDF by RP-HPLC has been reported[4,7]. Lamivudine (LMI), (2R,cis)-4-amino-1-(2-(hydroxylmethyl-1,3-oxathiolan-5-yl)-(1H) pyrimidin-2-one, is nucleoside-reverse transciptase inhibitor (NRTI)[1,2]. It is an analogue of cytidine. The estimation of lamivudine using UV[3,8–10] spectroscopy and HPLC has been reported[7,11].

Although the combination of EFV, TDF and LMI is not available commercially in the market, it is in phase 3 clinical trial and the safety and efficacy of TDF in combination with LMI and FFV has already been reported[12,13]. This study revealed that once daily regimen containing EFV, TDF and LMI is virologically and immunologically effective, well tolerated and safe with benefits in the lipid profile in the majority of patients. Hence the objective of the work is to develop new spectophotometric methods for estimating EFV, TDF and LMI in pharmaceutical formulation with good accuracy, simplicity, precision and economy.

UV double beam spectrophotometer (Shimadzu Model 1700) was employed with a spectral bandwidth of 1 nm and a wavelength accuracy of 0.3 nm (with automatic wavelength correction with a pair of 1 cm matched quartz cells). Pure samples of EFV, TDF and LMI and tablets (Label claim: EFV 600 mg, TDF 300 mg, LMI 300 mg) were obtained as gift samples from Ranbaxy Laboratories Ltd, Paonta Sahib, District-Sirmour, Himachal Pradesh, India. All the chemical and reagents used were of HPLC grade and purchased from spectrochem, Mumbai, India.

EFV, TDF and LMI (100 mg each) were separately weighed and transferred to a 100 ml volumetric flask and all the three drugs were dissolved in a mixture of methanol:water (50:50) to get a solution of 100 μg/ml. Working standard solutions of 20 μg/ml of each of the drugs were prepared and scanned in the range 400-200 nm to obtain the absorbance spectra and overlain spectra (fig. 1).

**Fig. 1:**
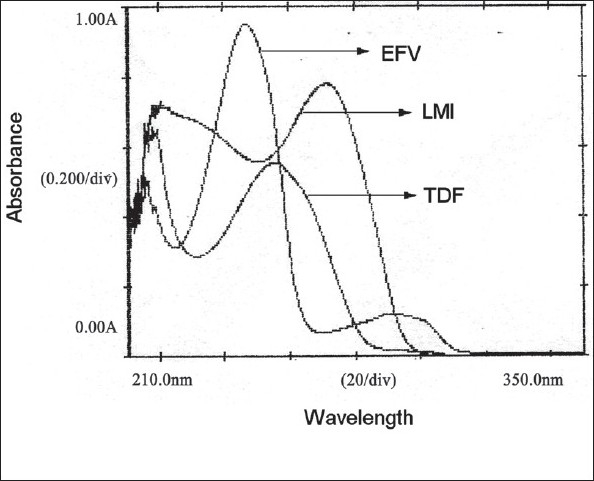
Overlain spectra of EFV, TDF and LMI Individual absorbance spectrum of EFV, TDF and LMI at 20 μg/ml concentration and overlain spectra

In the method I, three wavelengths 247, 259 and 272 nm were selected which are the λ_max_ of three drugs for the development of the simultaneous equations. The absorbances of EFV, TDF and LMI were measured and the absorptivity values E (1%, 1cm) were determined at all the three selected wavelengths. The concentrations of three drugs in mixture can be calculated using the following Eqns, C_EFV_= (A1(ay2az3-az2ay3)-ay1(A2az3-az2A3)+az1(A2ay3-ay2A3)/ax1(ay2az3-az2ay3)-ay1(ax2az3-az2ax3)+az1(ax2ay3-ay2ax3)…(1), C_TDF_= (ax1(A2az3-az2A3)-A1(ax2az3-az2ax3)+az1(ax2A3-A2ax3)/ax1(ay2az3-az2ay3)-ay1(ax2az3-az2ax3)+az1(ax2ay3-ay2ax3)…(2), and C_LMI_= (ax1(ay2A3-A2ay3)-ay1(ax2A3-A2ax3)+A1(ax2ay3-ay2ax3)/ax1(ay2az3-az2ay3)-ay1(ax2az3-az2ax3)+az1(ax2ay3-ay2ax3)…(3), where, C_EFV,_C_TDF_ and C_LMI_ are the concentrations of EFV, TDF and LMI, respectively in mixture and in sample solutions, A_1_, A_2_and A_3_ are the absorbances of sample at 247, 259 and 272 nm, respectively, ax_1_, ax_2_and ax_3_ are the absorptivity of EFV at 247, 259 and 272, respectively, ay_1_, ay_2_ and ay_3_ are the absorptivity of TDF at 247, 259 and 272 nm, respectively, az_1_, az_2_ and az_3_ are the absorptivity of LMI at 247, 259 and 272 nm, respectively.

In the method II, five mixed standards of EFV, TDF and LMI in the concentrations ratio of 10:5:5, 20:10:10, 30:15:15, 40:20:20 and 50:25:25 (μg/ml) were prepared by appropriate dilution of the standard stock solutions and scanned in the region of 400 to 220 nm in the multi-component mode using the four sampling wavelengths 247, 259, 260 and 272 nm, respectively. Recording of the absorbance of the mixed standard solutions processed by the instrument by means of matrix equations and then corrected to determine the concentrations of all the drugs in the tablet sample solutions (Table 1). The multicomponent overlain spectrum of EFV, TDF and LMI is shown in (fig. 2).

**TABLE 1:** RESULT OF ANALYSIS OF TABLET FORMULATION

	Method-I			Method-II			Method-III		
Parameters	EFV	TDF	LMI	EFV	TDF	LMI	EFV	TDF	LMI
Label claim	600	300	300	600	300	300	600	300	300
Drug content	100.05	100.15	100.07	100.10	100.08	100.13	100.01	100.02	100.13
SD	0.1695	0.4040	0.2821	0.1663	0.1833	0.1738	0.0248	0.0687	0.2557
%COV	0.1694	0.4033	0.2819	0.1661	0.1831	0.1735	0.0247	0.0686	0.2553
SE	0.0692	0.1649	0.1151	0.0678	0.0747	0.0708	0.0100	0.0280	0.1042

Value for drug content (%) is the mean of five estimations, SD is standard deviation, COV coefficient of variance, SE, standard error, Method I: Simultaneous equation method, Method II: Multi-wavelength spectroscopy, Method III: Derivative spectroscopy

**Fig. 2:**
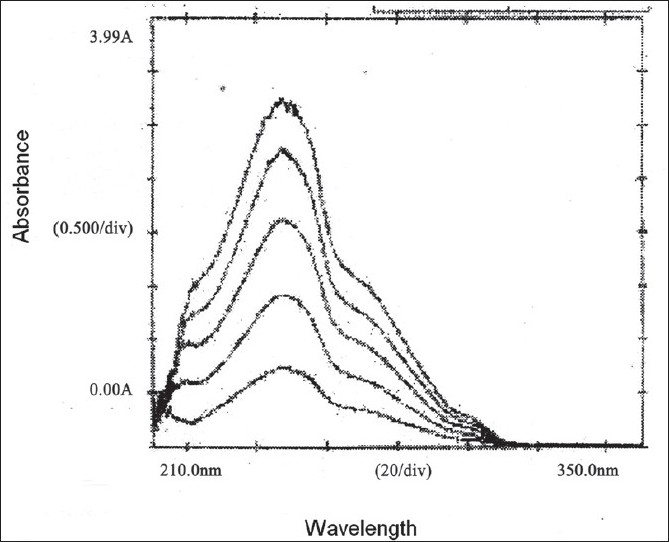
Overlain spectra of mixed standard of EFV, TDF and LMI Multi-component overlain spectra of five mixed standards in the concentration ratio of 10:5:5, 20:10:10, 30:15:15, 40:20:20 and 50:25:25 (μg/ml) of EFV, TDF and LMI, respectively.

In the third method, standard stock solutions of EFV, TDF and LMI were scanned from 200 to 400 nm. The spectra obtained were derivatised in first order and then overlain spectra recorded (fig. 3). From the entire derivative spectra obtained, the wavelengths selected in a manner such that EFV had Zero crossing point at 291.4 and 271.4 nm and TDF and LMI showed a measurable dA/dλ, TDF had the Zero crossing point at 291.4 and 305.6 and EFV and LMI showed a measurable dA/dλ. Whereas the Zero crossing point of LMI at 271.4 and 305.6 nm, EFV and TDF showed an appreciable dA/dλ. Hence, wavelengths 305.6, 291.4 and 271.4 nm were selected as analytical wavelengths for determination of EFV, TDF and LMI, respectively. Calibration curves were plotted between amplitudes observed at 1^st^ order (n=1), for three drugs at all the three wavelengths against the concentration, in the range of 10–60, 5–30 and 5–30 μg/ml for EFV, TDF and LMI, respectively. Estimation of these drugs was done by solving the regression equations, i.e. for EFV y= 0.0006x+0.0003, for TDF y= 0.0014x+(-0.0005), For LMI, y=0.0002x+(-0.0013).

**Fig. 3:**
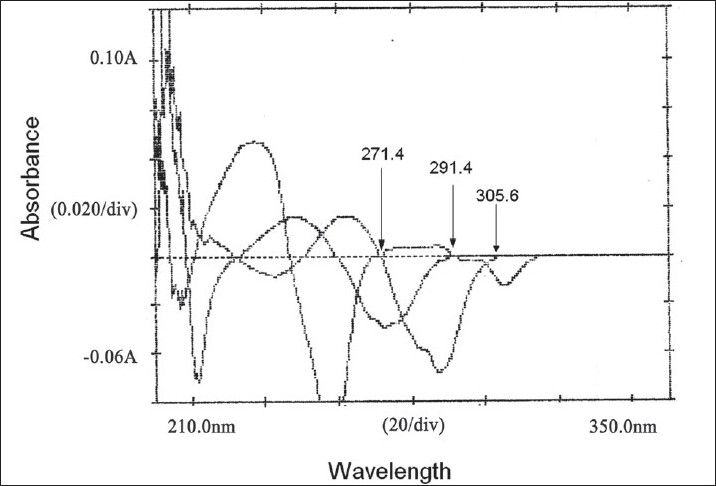
First order derivative overlain spectra of EFV, TDF and LMI Zero crossing points of LMI (305.6 nm), TDF (271.4nm) and LMI (291.4 nm)

For analysis of tablets, 20 tablets were weighed individually and their average weight was determined. The tablets were then crushed to fine powder and powder equivalent to the weight of one tablet was transferred to 100 ml volumetric flask and dissolved in 50 ml of mixture of methanol:water (50:50) for 10 min with vigorous shaking. Finally, the volume was made up to the mark with solvent system. The solution was then filtered through Watmann filter paper (# 41). Then volume was made up to the mark with solvent system. The concentration of each analyte was determined using the Eqns. generated in method I and method III. In method II, analysis of the resulting solution was carried out using the multicomponent mode of the instrument (Table 1).

To check the accuracy of the developed methods and to study the interference of formulation additives, analytical recovery experiments were carried out by the standard addition method. The means of % recovery (% COV) were found to be < 1.0 for three methods (Table 2).

**TABLE 2:** RESULT OF RECOVERY STUDY OF EFV, TDF AND LMI

Method	Amount taken (mg/ml)			Amount added %	% recovery			%COV		
	EFV	TDF	LMI		EFV	TDF	LMI	EFV	TDF	LMI
I	30	15	15	80	100.13	100.30	100.08	0.0665	0.1886	0.4196
	30	15	15	100	100.17	100.39	100.17	0.2354	0.8809	0.8809
	30	15	15	120	98.96	100.22	100.09	0.0254	0.3599	0.2813
II	30	15	15	80	100.10	100.05	100.12	0.1733	0.0584	0.1624
	30	15	15	100	100.06	100.07	100.15	0.1222	0.2157	0.2169
	30	15	15	120	99.99	100.09	100.17	0.1123	0.1049	0.2889
III	30	15	15	80	100.16	100.05	100.19	0.1579	0.0641	0.2696
	30	15	15	100	100.06	100.20	100.23	0.0320	0.2565	0.3326
	30	15	15	120	98.97	100.11	100.01	0.0152	0.1663	0.1946

% recovery is the mean of three estimations, Method I: Simultaneous equation method, Method II: Multi-wavelength spectroscopy, Method III: Derivative spectroscopy, COV: Coefficient of variance.

To check the degree of repeatability of the methods, suitable statistical evaluation was carried out. Five samples of the tablet formulations were analyzed for the repeatability study. The standard deviation (SD), coefficient of variance (COV), and standard error (SE) were calculated (Table 1).

The concentrations of three drugs were measured three times on the same day at intervals of 1 h and on three different days for intra and interday study, respectively. The limits of detection and quantitation, LOD and LOQ, were calculated by use of the equations LOD = 3.3σ/S and LOQ = 10σ /S, where σ is the standard deviation of the blank and S is the slope of the calibration curve (Table 3).

**TABLE 3:** INTRADAY, INTERDAY, LOD AND LOQ DATA FOR EFV, TDF, AND LMI

Method	Drug	Intraday precision % COV (n = 3)	Interday precision % COV			LOD (μg/ml)	LOQ (μg/ml)
			Day 1	Day 2	Day 3		
Method I	EFV	0.49	0.1609	0.4618	0.8964	0.040	0.217
	TDF	0.5071	0.2929	0.5095	0.8475	0.894	0.127
	LMI	0.856	0.7484	0.2525	0.5170	0.154	1.144
Method II	EFV	0.4618	0.2245	0.2160	0.6333	0.072	0.618
	TDF	0.2283	0.1098	0.3495	0.7449	0.861	0.148
	LMI	0.5346	0.0936	0.2231	0.3550	0.158	0.144
Method III	EFV	0.6347	0.2610	0.6496	0.5847	0.031	0.095
	TDF	0.1676	0.2778	0.3111	0.8142	0.202	0.613
	LMI	0.5388	0.1601	0.2231	0.3366	0.099	0.302

COV: Coefficient of variance, LOD: Limit of detection and LOQ: Limit of quantitation. Method I: Simultaneous equation method, Method II: Multi-wavelength spectroscopy and Method III Derivative spectroscopy.

The linear regression equations obtained were: absorbance at 247 nm=0.0472x+0.0631 (For EFV, r^2^=0.9993), at 259 nm=0.0199x+0.068 (For TDF, r^2^=0.9997), at 272 nm=0.0398x+0.0275 (r^2^=0.9998) for simultaneous equation method and at 305.6 nm=0.0006x+0.0003 (For EFV, r^2^=0.9992), at 271.4 nm=0.0014 x+(-0.0005) (For TDF, r^2^=0.9995), at 291.4 nm=0.002x+(-0.0013) (r^2^=0.9992) for derivative spectroscopy method, respectively.

The assay value of EFV, TDF and LMI for method-I was found to be 100.05, 100.15, 100.07, respectively, with a standard deviation of < 1.0; the assay value for, method-II was found to be 100.10, 100.08 and 100.13, respectively, with standard deviation of < 1.0 the assay value for method-III was found to be 100.01, 100.02 and 100.13, respectively, with a standard deviation of < 1.0 (Table 1).

Assay values of formulation were the same as mentioned in the label claim, indicating that the inference of the excipient matrix is insignificant in estimation of EFV, TDF and LMI by three proposed methods. The proposed methods were found to be accurate, precise, reproducible and stable, and can be successfully applied for the routine analysis of all the three drugs in combined tablet dosage forms.

## References

[1] Budawari S (2001). The Merck Index.

[2] Sweetman SC (2002). Martindale: The complete drug reference.

[3] Nagori BP, Kumar P (2008). Simultaneous spectrophotometric determination of Efavirenz and Lamivudine. Indian Drugs.

[4] Mangaonkar K, Desai A (2008). Simultaneous estimation of emtricitabine, tenofovir disoproxil fumarate and efavirenz from tablets by reverse phase high performance chromatography method. Indian Drugs.

[5] Sentenac S, Fernandez C, Thuillier A, Lechat P, Aymard G (2001). Sensitive determination of tenofovir in human plasma samples using reversed-phase liquid chromatography. J Chromatogr B Analyt Technol Biomed Life Sci.

[6] Kandagal PB, Manjunatha DH, Seetharamappa J, Kalanur SS (2008). RP-HPLC method for the determination of tenofovir in pharmaceutical formulations and spiked human plasma. Anal Lett.

[7] Mangaonkar K, Desai A (2008). Simultaneous estimation of lamivudine and tenofovir disoproxil fumarate in tablets by isocratic reverse phase high performance liquid chromatography method. Indian Drugs.

[8] Uslu B, Özkan SA (2002). Determination of lamivudine and zidovudine in binary mixtures using first derivative spectrophotometric, first derivative of the ratio-spectra and high-performance liquid chromatography-UV methods. Anal Chim Acta.

[9] Kapoor N, Khandavilli S, Panchagnula R (2006). Simultaneous determination of lamivudine and stavudine in antiretroviral fixed dose combinations by first derivative spectrophotometry and high performance liquid chromatography. J Pharm Biomed Anal.

[10] Sankar G, Reddy MV, Rajendra Kumar JM, Murthy TK (2002). Spectrophotometric determination of lamivudine and stavudine. Indian J Pharm Sci.

[11] Palled MS, Rajesh PM, Chatter M, Bhat AR (2005). Reverse phase high performance liquid chromatographic determination of ziduvudine and lamivudine in tablet dosage form. Indian J Pharm Sci.

[12] Cassetti JV, Madruga JM, Suleiman A, Etzel L, Zhong AK, Cheng J (2007). Safety and efficacy of tenofovir DF in combination with lamivudine and efavirenz through 6 years in antiretroviral-naïve HIV-1-infected patients. HIV Clin Trials.

[13] Arrizabalaga J, Arazo P, Aguirrebengoa K, García-Palomo D, Chocarro A, Labarga P (2007). Unit of infectious diseases, Hospital Donostia, Donostia-San Sebastián, Spain. HIV Clin Trials.

